# Development of a dense genetic map and QTL analysis for pod borer *Helicoverpa armigera* (Hübner) resistance component traits in chickpea (*Cicer arietinum L*.)

**DOI:** 10.1002/tpg2.20071

**Published:** 2020-12-01

**Authors:** Rutwik Barmukh, Manish Roorkiwal, Jagdish Jaba, Annapurna Chitikineni, Suraj Prasad Mishra, Someswar Rao Sagurthi, Rajendra Munghate, HC Sharma, Rajeev K. Varshney

**Affiliations:** ^1^ Center of Excellence in Genomics & Systems Biology International Crops Research Institute for the Semi‐Arid Tropics (ICRISAT) Hyderabad India; ^2^ Department of Genetics Osmania University Hyderabad India; ^3^ Theme‐Integrated Crop Management International Crops Research Institute for the Semi‐Arid Tropics (ICRISAT) Hyderabad India

## Abstract

Genetic enhancement for resistance against the pod borer, *Helicoverpa armigera* is crucial for enhancing production and productivity of chickpea. Here we provide some novel insights into the genetic architecture of natural variation in *H. armigera* resistance in chickpea, an important legume, which plays a major role in food and nutritional security. An interspecific recombinant inbred line (RIL) population developed from a cross between *H. armigera* susceptible accession ICC 4958 (*Cicer arietinum*) and resistant accession PI 489777 (*Cicer reticulatum*) was evaluated for *H. armigera* resistance component traits using detached leaf assay and under field conditions. A high‐throughput Axiom*CicerSNP* array was utilized to construct a dense linkage map comprising of 3,873 loci and spanning a distance of 949.27 cM. Comprehensive analyses of extensive genotyping and phenotyping data identified nine main‐effect QTLs and 955 epistatic QTLs explaining up to 42.49% and 38.05% phenotypic variance, respectively, for *H. armigera* resistance component traits. The main‐effect QTLs identified in this RIL population were linked with previously described genes, known to modulate resistance against lepidopteran insects in crop plants. One QTL cluster harbouring main‐effect QTLs for three *H. armigera* resistance component traits and explaining up to 42.49% of the phenotypic variance, was identified on CaLG03. This genomic region, after validation, may be useful to improve *H. armigera* resistance component traits in elite chickpea cultivars.

## Core Ideas

- A dense genetic map with 3,873 SNP markers, and spanning a distance of 949.27 cM was developed.
- Nine main‐effect QTLs and 955 epistatic QTLs explaining up to 42.49% and 38.05% PVE, respectively, for *Helicoverpa armigera* resistance component traits were identified.
- A QTL cluster on CaLG03 holds great potential to improve *Helicoverpa armigera* resistance component traits in chickpea.
- The identified QTLs encompassed some genes that were previously described to modulate resistance against lepidopteran insects.

**Abbreviations**
CaLG: *Cicer arietinum* linkage group
E‐QTL: Epistatic quantitative trait locus
GAB: Genomics‐assisted breeding
GMM: Genotype matrix mapping
GO: Gene ontology
ICRISAT: International Crops Research Institute for the Semi‐Arid Tropics
LOD: Logarithm of odds
MeSA: Methyl salicylate
M‐QTL: Main‐effect quantitative trait locus
NGS: Next‐generation sequencing
PCA: Principal component analysis
PVE: Phenotypic variance explained
QTL: Quantitative trait locus
RIL: Recombinant inbred line
SNP: Single nucleotide polymorphism.

## 1 INTRODUCTION

Chickpea (*Cicer arietinum* L.) is one of the most important food legume crops cultivated on marginal lands in more than 56 countries and contributes considerably to the food and nutritional security. This crop is currently grown on over 17.81 million hectares with a production of 17.19 million tonnes, of which Asia accounts for 77% production (FAOSTAT, 2018). Although chickpea production has witnessed continuous growth over the last few decades, the crop cultivars are sensitive to multiple biotic and abiotic factors (Muehlbauer & Sarker, 2017). Being an abundant resource of proteins, chickpea is severely damaged by a wide range of insect pests in the field as well as during storage (Clement et al., 2000). Among multiple pests damaging this crop, the pod borer, *Helicoverpa armigera* (Hübner), which is also the single most key yield reducing factor in food legumes, causes an estimated loss of US$ 328 million in chickpea. Worldwide, it causes a projected economic loss of about US $2 billion annually, despite using insecticides costing over US $1 billion to control this pest (Sharma, Varshney, Gaur, & Gowda, 2008). In general, the estimates of yield losses vary from 50–100% in the tropics, and 5–10% in the temperate regions (Patil, Goyal, Chitgupekar, Kumar, & El‐Bouhssini, 2017). In accordance with several widely grown staple crops, domestication in chickpea has resulted in a narrow genetic base. Breeding efforts over the last six decades have also been limited to introduce diverse germplasm at the desired level (Varshney, Thudi, May, & Jackson, 2010). With the anticipated climate change, there is a rising demand to develop improved chickpea varieties with a higher yield, and disease and pest resistance to meet the needs of the burgeoning population throughout the world.

The pod borer can infest chickpea plants at any growth stage, from seedling to maturity and damage multiple lateral branches of the plant including leaves, flowers and pods. The neonate larvae (1^st^ and 2^nd^ instar) initially feed on leaves and tender twigs of the plant during the vegetative stage, while the latter stage larvae (3^rd^, 4^th^ and 5^th^ instar) feed on leaves and mature pods (Patil et al., 2017). Feeding on flower buds and flowers reduces fruit setting that causes up to 50% yield losses (Reed & Pawar, 1982). Management of *H. armigera* is mostly dependent on insecticide applications; however, their indiscriminate use has resulted in the development of insecticide resistance, pest resurrection and damage to other non‐target useful organisms. As a result, in order to avoid or delay the development of resistance to insecticides, breeding of pod borer‐resistant chickpea varieties is considered to be the most effective and sustainable management approach. In this regard, a range of genetic resources have been screened; however, no source possessing very high levels of resistance against *H. armigera* has been identified. Some lines with partial resistance have been reported in the cultivated germplasm and have been used in chickpea breeding (Sharma, 2001; Sharma, Pampapathy, Lanka, & Ridsdill‐Smith, 2005; Singh, Sharma, Varshney, Sharma, & Singh, 2008). Moderate levels of resistance against *H. armigera* have been observed in some accessions of the wild species including *C. reticulatum, C. echinospermum*, *C. judaicum*, and *C. pinnatifidum* (Golla, Rajasekhar, Sharma, Hari Prasad, & Sharma, 2018; Sharma et al., 2005). *C. reticulatum* is compatible for crossing with *C. arietinum* and therefore identification of QTLs for *H. armigera* resistance from *C. reticulatum* species will be very useful for chickpea breeding. The RIL population derived from ICC 4958 (*C. arietinum*) × PI 489777 (*C. reticulatum*) is considered as the reference mapping population and has been used for linkage mapping in several studies using various marker systems (Gaur et al., 2015; Gaur, Verma, Pradhan, Ambreen, & Bhatia, 2020; Gujaria et al., 2011; Thudi et al., 2011). Understanding the genetic architecture and identification of closely linked markers for *H. armigera* resistance component traits, in the above‐mentioned RIL population, will be useful in deploying molecular breeding for pyramiding pod borer resistance genes in a faster and precise way.

**Core Ideas**

- A dense genetic map with 3,873 SNP markers, and spanning a distance of 949.27 cM was developed.
- Nine main‐effect QTLs and 955 epistatic QTLs explaining up to 42.49% and 38.05% PVE, respectively, for *Helicoverpa armigera* resistance component traits were identified.
- A QTL cluster on CaLG03 holds great potential to improve *Helicoverpa armigera* resistance component traits in chickpea.
- The identified QTLs encompassed some genes that were previously described to modulate resistance against lepidopteran insects.

Recent innovations in next‐generation sequencing (NGS) and high‐throughput genotyping capabilities have enabled the use of germplasm resources to capture several million variations throughout the genome in a cost‐effective manner. For instance, draft genome sequence have become available in several pulse crops, such as chickpea (Varshney et al., 2013), pigeonpea (Varshney et al., 2012a), adzuki bean (Kang et al., 2015) and pea (Kreplak et al., 2019). Moreover, germplasm sequencing efforts have facilitated the identification of a large number of single nucleotide polymorphism (SNP) markers to detect genomic loci/candidate genes underlying complex traits in chickpea (Varshney et al., 2019). In order to utilize these markers in routine breeding programs, there is an emerging need to develop cost‐effective high‐throughput genotyping platforms. To overcome this problem, the recently developed Axiom*CicerSNP* array holds great potential to detect genome‐wide SNPs in a high‐throughput manner for the development of dense linkage maps (Roorkiwal et al., 2018).

With an objective to dissect *H. armigera* resistance into component traits, apprehend the underlying genetic basis and identify candidate genes for diverse component traits, the present study undertakes generation of phenotyping and genotyping data on an inter‐specific RIL population (ICC 4958 × PI 489777). We report here the development of a dense genetic map, and identification of both main‐effect and epistatic QTLs for diverse *H. armigera* resistance component traits. The QTLs and candidate genes identified in this study, after validation, could be useful for deploying molecular breeding to develop superior chickpea varieties with improved *H. armigera* resistance component traits.

## 2 MATERIALS AND METHODS

### 2.1 Plant material and phenotypic evaluation

A chickpea RIL population generated from a cross between ICC 4958 (*H. armigera* susceptible genotype) and PI 489777 (*H. armigera* resistant genotype), known as CRIL 2 internationally, was used in this study. A total of 124 RILs developed using single‐seed descent method were used for phenotyping data collection for a total of 12 *H. armigera* resistance component traits, and for genetic analyses.

The RIL population was bio‐assayed for *H. armigera* resistance under no‐choice conditions using detached leaf assay (Sharma et al., 2005). The plants were grown under controlled conditions in plastic pots that were filled with a potting mixture and farmyard manure in the ratio of 2:1. The larvae of *H. armigera* used in bioassays were reared in the Entomology Laboratory at ICRISAT, Patancheru, India. The *H. armigera* larvae were reared individually on chickpea based artificial diet (Babu et al., 2014) at 25 ± 2 °C, 60–70% relative humidity, and 16:8 h (L/D) photoperiod regime. During vegetative and flowering stages, the terminal leaf branches were screened for their resistance to second instar larvae of *H. armigera* using a detached leaf assay. Here, 10 millilitres of boiled agar‐agar (3%) was poured into plastic cups (4.5 × 11.5 cm diameter) kept in a slanting manner. A terminal branch with 3–4 fully expanded leaves and a terminal bud was cut with a sharp knife and immediately placed inside the cup in a slanting manner into agar‐agar medium, which was kept in the laboratory at 25 ± 2 ° C and 60–70% relative humidity (Jaba, Agnihotri, & Chakravarty, 2017a; Sharma et al., 2005). Consequently, 10 neonate *H. armigera* larvae were released on the chickpea leaves in each replication, and the cup was covered with a lid. The experiment was conducted using up to three replications in a completely randomized design. Observations were made at five days after the release of larvae. In the first instance, the plants were rated for leaf damage on a scale of 1 to 9 (where 1 = < 10% leaf area damaged and 9 = > 80% leaf area damaged). The total number of surviving larvae after the feeding period were recorded, and their weights were measured pre‐ and post‐feeding. The data were represented as the larval survival percentage and mean weight of the larvae. In brief, five *H. armigera* resistance component traits were evaluated using detached leaf assay, which include damage rating at vegetative stage (DRV_L), damage rating at flowering stage (DRF_L), unit larval weight at vegetative stage (ULWV), unit larval weight at flowering stage (ULWF) and larval survival percentage (LSP).

In addition, the RILs were also screened under field conditions at ICRISAT, India, in two replications during 2007–2008 and 2008–2009 post‐rainy seasons. The plot size included four rows of 2 m length with a row‐to‐row spacing of 60 cm and plant‐to‐plant distance of 10 cm within a row. Observations were recorded on the larval population of *H. armigera* from five plants that were selected at random from each plot. The plants were tagged, and the larval count was recorded during the vegetative and flowering stages of the crop. Moreover, the overall resistance score after *H. armigera* damage during the vegetative and flowering stages was also recorded. Here, the plants were visually scored for damage due to leaf feeding on a 1 to 9 scale. The percent pod damage by the pod borer, *H. armigera* was estimated from tagged plants at the time of harvest to assess the extent of the damage. A total of 100 pods were gathered from each replication, and all the pods were critically assessed for the damage of *H. armigera* (Jaba, Devrani, Agnihotri, & Chakravarty, 2017b). Number of healthy and damaged pods due to pod borer were recorded separately for each sample and converted into percentage pod damage value. Phenotyping data was also documented for phenological traits, including days to first flowering and days to 50% flowering. Days to 50% flowering were measured as the number of days from sowing up to 50% plants in a plot producing their first flower. Taken together, seven component traits were evaluated under field conditions, which include damage rating at vegetative stage (DRV_F), pod damage percentage (PDP), larvae per 10 plants (LPP), number of larvae per plant at vegetative stage (NLV), number of larvae per plant at flowering stage (NLF), days to first flowering (DFF) and days to 50% flowering (D50F).

### 2.2 Statistical analyses

The phenotypic variation observed in the mapping population for *H. armigera* resistance component traits was analysed using GenStat software version 15.0 (VSN International Ltd., Hemel Hempstead, UK). Analysis of differences between genotype means was evaluated using the Tukey‐Kramer test (0.05 significance level). The ‘UsingR’ package within R version 3.6.1 (R Project for Statistical Computing) was used for the analysis and plotting of normal histograms with a frequency distribution curve. A Pearson correlation analysis was performed with custom R script using ‘Hmisc’ package, and the correlation heatmap was constructed using ‘pheatmap’ package in R. A principal component analysis factor graph, used for visualizing the relationship between multiple traits, was computed using ‘FactoMineR’ package in R environment.

### 2.3 DNA extraction and genotyping using Axiom*CicerSNP* array

DNA was extracted from the fresh chickpea leaves of RILs and parental genotypes using a high‐throughput mini‐DNA extraction method, as described previously (Jain et al., 2019). The quality and quantity of DNA was estimated using a spectrophotometer (Shimadzu UV160A, Japan). The high‐quality DNA extracted was used for genotyping using the high‐throughput Axiom*CicerSNP* array. In summary, the isolated DNA (100 ng) was fragmented (25–125 bp) and purified before being hybridized with the genotyping platform. Further, the non‐specific binding of random fragments at the array surface was washed off, and the GeneTitan Multi‐Channel instrument was utilized to generate the data. The Axiom Analysis Suite (1.1.0.616) was employed for allele calling by using the ‘*.CEL files*’. High‐quality genotyping results were obtained by following the Axiom array protocol mentioned in Roorkiwal et al. (2018).

### 2.4 Data filtering

High‐quality genotyping data was compiled for 50,590 SNP loci, spanning the entire chickpea genome. Since RIL population should be entirely homozygous, the heterozygous calls obtained for SNP markers were considered as missing data. Initially, SNPs displaying contrasting alleles for parental genotypes were selected. Subsequently, a chi‐square test was performed for all the SNPs with a null hypothesis that two alleles at a particular locus segregate in a 1:1 ratio for the RIL population. The SNPs showing marked differences from the 1:1 ratio were filtered out from the further analysis. We tested the resulting SNPs and removed SNPs possessing more than 15% missing data. Also, genotypes containing greater than 30% missing genotypic data and more than 90% identity to another RIL were eliminated. The final QTL analysis was performed using 3,873 SNP markers from 90 independent lines of the ICC 4958 × PI 489777 RIL population.

### 2.5 Genetic map construction

A genetic map was constructed using JoinMap version 4.0 (Van Ooijen & Voorrips, 2006). The maximum‐likelihood algorithm and Kosambi mapping function using the pre‐set default settings were used for linkage mapping analysis. Map calculations for constructing the linkage map were conducted using a logarithm of odds (LOD) score of 3.0, initial acceptance probability of 2.5, and a chain length of 500 per Monte Carlo EM cycle was used for estimation of recombination frequencies. Placement of markers within diverse linkage groups was performed using the commands ‘LOD grouping’ and ‘Create groups using the groupings tree’. The computed linkage map was represented using MapChart version 2.2 (Vorrips, 2002).

### 2.6 QTL analysis

The QTL analysis was conducted using the R/qtl package (Broman, Wu, En, & Churchill, 2003) within the R statistical computing environment. In the first instance, QTL mapping was conducted using simple interval mapping by implementing the Haley‐Knott regression algorithm (Haley & Knott, 1992). The empirical LOD threshold was calculated using 10,000 permutations (at α = 0.05 significance level) that led to a LOD score of 2.8. Confidence intervals were computed using the 1.5‐LOD drop method. The ggplot2 package in R was deployed for generating good quality QTL graphs (Wickham, 2009). The interaction between two peaks was computed using “scantwo” function in R/qtl (Haley‐Knott regression) and the significance of this interaction was estimated using 10,000 permutations (at α = 0.05). The phenotypic variance of the observed peaks was calculated using a single peak model.

The epistatic interaction analysis for identifying interacting QTLs was performed using the Genotype Matrix Mapping software (GMM; v 2.1, https://www.kazusa.or.jp/GMM). The combinations of two loci and three loci interactions were computed using GMM, as described previously (Sivashakthi et al., 2018). Here, the syntax ‘AA’ and ‘BB’ indicate alleles contributed by the parental genotypes ICC 4958 and PI 489777, respectively.

### 2.7 Candidate gene identification

The amino acid sequences anticipated from the gene models of candidate genes present within the identified QTL intervals were retrieved from the draft genome sequence (CaGAv1.0) of chickpea (Varshney et al., 2013). These sequences were searched against NCBI‐nr protein database using BLAST program in BLAST2GO software (Conesa et al., 2005). The gene ontology (GO) terms associated with the sequences were searched for plant‐related GO terms, as described previously (Jaganathan et al., 2015).

## 3 RESULTS

### 3.1 Phenotypic variation in the RIL population

The inter‐specific RIL mapping population (ICC 4958 × PI 489777) was phenotyped for a total of 12 *H. armigera* resistance component traits for up to two seasons at ICRISAT, India (Table 1). The significant features of component traits and statistical analyses, such as mean performance of parents, variations in RILs, broad‐sense heritability (*H* ^2^) and source of variations for traits phenotyped using the RIL population are provided in Supplemental Table S1.

**TABLE 1:** Summary of *H. armigera* resistance component traits, trait codes, units, and season of phenotyping

Trait name	Code (unit)	Season of phenotyping
***Detached leaf assay***		
Damage rating at vegetative stage	DRV_L	2007–2008 and 2008–2009
Damage rating at flowering stage	DRF_L	2007–2008
Unit larval weight at vegetative stage	ULWV (mg)	2007–2008 and 2008–2009
Unit larval weight at flowering stage	ULWF (mg)	2007–2008
Larval survival percentage	LSP (%)	2008–2009
***Field evaluation***		
Damage rating at vegetative stage	DRV_F	2007–2008 and 2008–2009
Pod damage percentage	PDP (%)	2008–2009
Larvae per 10 plants	LPP	2007–2008
Number of larvae per plant at vegetative stage	NLV	2008–2009
Number of larvae per plant at flowering stage	NLF	2008–2009
Days to first flowering	DFF	2008–2009
Days to 50% flowering	D50F	2008–2009

The RIL population was characterized using detached leaf assay in the laboratory and under field conditions for *H. armigera* resistance component traits. A continuous variation was observed for all the traits measured using detached leaf assay (Supplemental Figure S1) and through field evaluation (Supplemental Figure S2). Moreover, the phenotypic trait value of multiple RILs surpassed their parents in both directions for all evaluated traits, which indicates transgressive segregation in the RIL population (Supplemental Figure S1, S2). A long‐range variation in leaf damage rating was observed at flowering stage (DRF_L), in which some lines had a damage score of 2.0 while others had a damage score as high as 8.0; with a *H* ^2^ of 47.69%. A considerable variation in days to first flowering (DFF) was observed under field conditions, in which some lines flowered as early as 26 days, while some other flowered as late as 108 days after sowing; with a high *H* ^2^ of 83.20%. In general, *H* ^2^ of the studied traits ranged from 8.03% to 92.87%. Analysis of variance results indicated that variations among the RILs were significant (at *p *< .001 or *p *< .01) for all the traits, except for damage rating at vegetative stage (2007–2008), unit larval weight at vegetative stage (2007–2008) and the number of larvae per plant at vegetative stage (Supplemental Table S1). More importantly, the environmental (seasonal) differences and genotype × environment interaction effects were also substantial for three traits evaluated across two seasons (Supplemental Table S2).

### 3.2 Correlations among component traits

**FIGURE 1:**
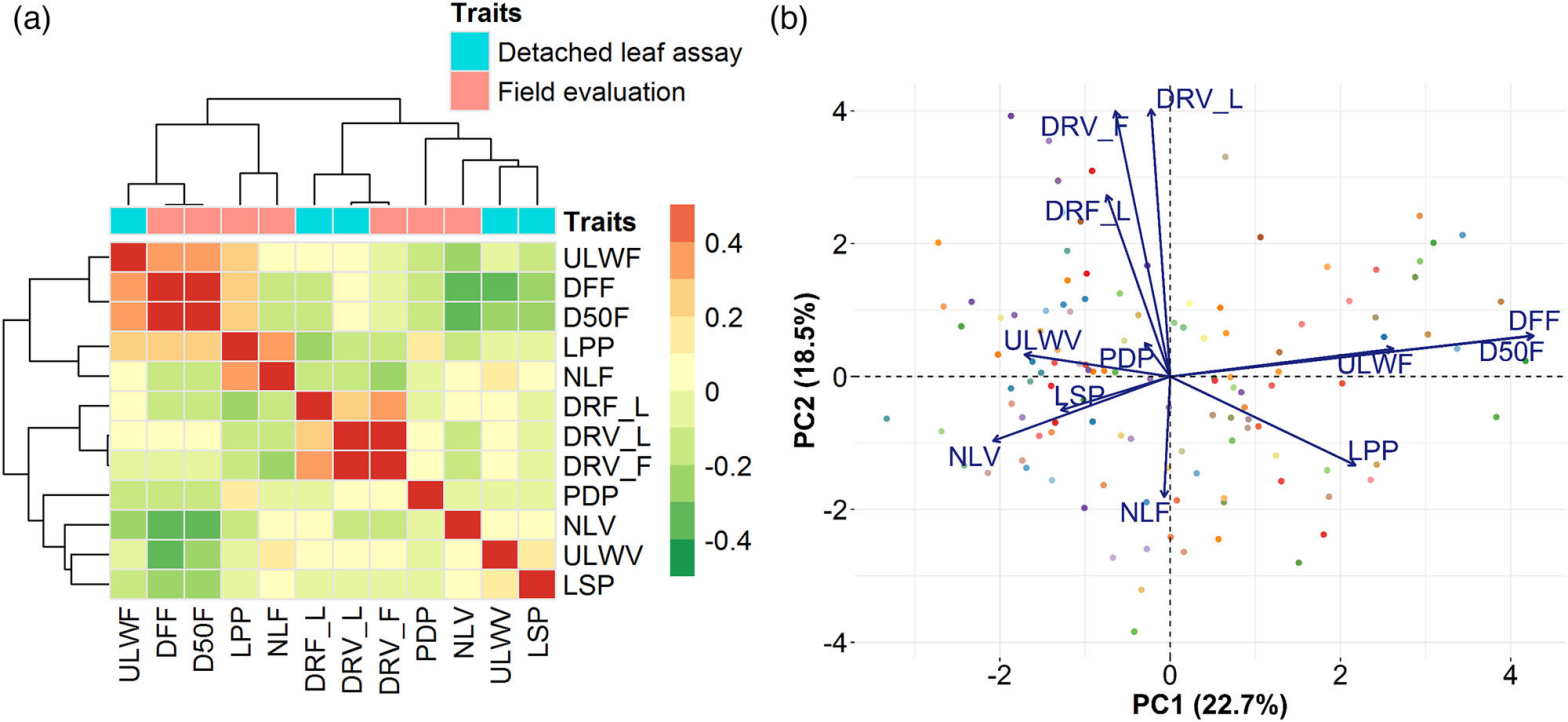
Correlation matrix and principal component analysis for deciphering the relationship between studied traits. Graphical representation of (a) Pearson correlation matrix and (b) principal component analysis (PCA) biplot is shown for *H. armigera* resistance component traits. In the PCA, positively correlated variables are grouped together, while negatively correlated variables are placed on the opposite side of the origin. Distance between variables and the origin reveals the quality of the variables on the factor map. Color dots represent different RILs and their position correspond to specific trait loadings with respect to PC1 and PC2

To determine the correlation between *H. armigera* resistance component traits measured using detached leaf assay and field evaluation, a Pearson correlation analysis was undertaken (Figure 1a; Supplemental Table S3). Damage rating at vegetative stage evaluated using detached leaf assay was significantly and positively correlated with damage rating at vegetative stage measured under field conditions. This significant positive correlation reaffirmed the potential of detached leaf assay in accurately determining pod borer damage similar to the field conditions, at initial growth stages. A positive correlation was observed between damage rating at vegetative stage and at flowering stage, both evaluated using detached leaf assay. Notably, the flowering response was negatively correlated with unit larval weight at vegetative stage, while it displayed a positive correlation with unit larval weight measured at flowering stage. A substantial positive correlation was observed between unit larval weight at flowering stage and larvae per 10 plants. In addition, a marked negative correlation was observed between flowering time and the number of larvae per plant at vegetative stage.

Furthermore, a principal component analysis (PCA) biplot provided an overview of the relationships between different *H. armigera* resistance component traits phenotyped under the laboratory and field conditions (Figure 1b). The first two principal components (PC1 and PC2) explained nearly 41% of the total variability. Here, an increase in leaf damage rating at flowering stage favoured pod damage. Days to first flowering and days to 50% flowering were closely linked to unit larval weight at flowering stage and distantly related to the number of larvae per plant at vegetative stage. This suggested that flowering time variation greatly influenced the growth and development of larvae feeding on chickpea plants. In addition, an increase in the number of larvae per plant at vegetative stage or at flowering stage favoured an upsurge in larval survival percentage.

### 3.3 A dense genetic linkage map

**FIGURE 2:**
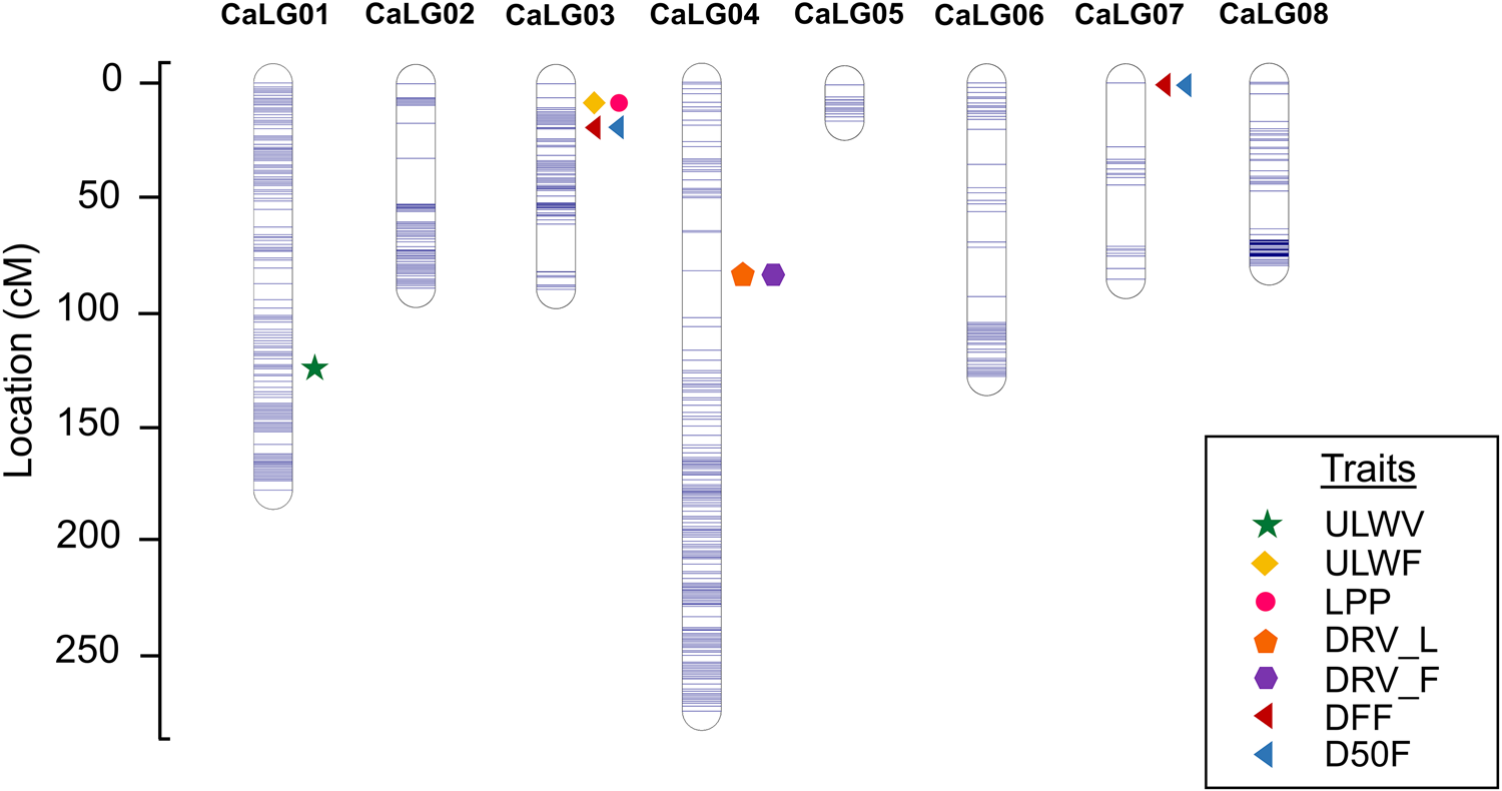
Graphical representation of the linkage map comprising of 3,873 SNP markers for ICC 4958 × PI 489777 RIL population. Main‐effect QTLs for seven traits are represented using diverse symbols, as depicted in the Traits Key. *cM*, centiMorgan

We utilized the genotypic data generated using the Axiom *CicerSNP* array for constructing a genetic map (Roorkiwal et al., 2018). High quality data for a total of 18,033 polymorphic SNPs was obtained, and used for further analysis. After several filtering steps (Supplemental Figure S3), a total of 3,873 SNPs covering the entire genome were effectively mapped to eight linkage groups (CaLG01 to CaLG08) spanning a distance of 949.27 cM (Table 2). A graphical representation of the predicted genetic map is provided in Figure 2, and a comparison of the physical map and genetic map is provided in Supplemental Figure S4. The highest number of markers (1,419 SNPs) were mapped on CaLG04, whereas CaLG05 contained the lowest number of markers (52 SNPs). Furthermore, the length of the linkage groups ranged from 15.85 cM for CaLG05 to 276.99 cM for CaLG04. The average marker density varied from as high as 5.40 SNPs/cM on CaLG03 to 0.95 SNPs/cM on CaLG07, with an average marker density of 3.62 SNPs/cM.

**TABLE 2:** Features of genetic map constructed for ICC 4958 × PI 489777 RIL population using Axiom*CicerSNP* array

Serial number	Linkage group	SNPs on Axiom*CicerSNP* array	Homozygous polymorphic SNPs	Number of SNPs mapped	Map distance (cM)	Inter‐marker distance (cM)	Density (SNPs/cM)
1.	CaLG01	8782	3014	945	179.44	0.19	5.27
2.	CaLG02	4435	1580	348	90.09	0.26	3.86
3.	CaLG03	3586	1295	489	90.59	0.19	5.40
4.	CaLG04	16773	6653	1419	276.99	0.20	5.12
5.	CaLG05	3579	1051	52	15.85	0.30	3.28
6.	CaLG06	6302	2248	338	129.19	0.38	2.62
7.	CaLG07	5244	1305	82	86.42	1.05	0.95
8.	CaLG08	1889	887	200	80.69	0.40	2.48
	Average	6323.75	2254.13	484.13	118.66	0.37	3.62
	Total	50590	18033	3873	949.27		

*SNPs*, single nucleotide polymorphisms; *cM*, centiMorgan.

### 3.4 QTLs for *H. armigera* resistance component traits

**FIGURE 3:**
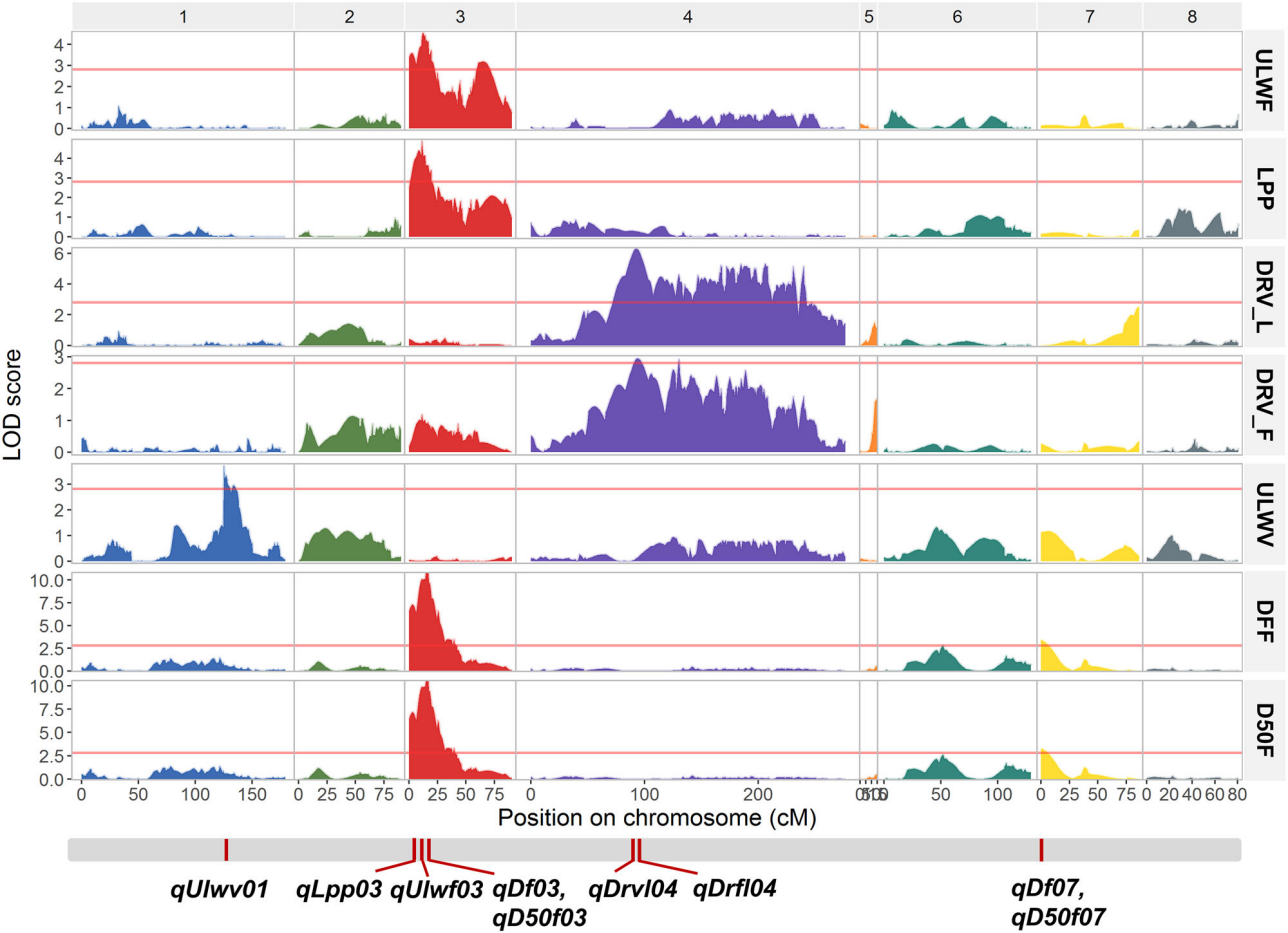
Genome‐wide distribution of main‐effect QTLs for seven *H. armigera* resistance component traits across chickpea genome. Nine main‐effect QTLs identified for seven *H. armigera* resistance component traits [unit larval weight at flowering stage (ULWF), larvae per 10 plants (LPP), damage rating at vegetative stage under detached leaf assay for 2007–2008 season (DRV_L), damage rating at flowering stage under detached leaf assay (DRF_L), unit larval weight at vegetative stage for 2008–2009 season (ULWV), days to first flowering (DFF) and days to 50% flowering (D50F)] have been displayed. The name ascribed to these main‐effect QTLs are provided at the bottom of the plot. The genetic distance (cM) is indicated on X‐axis and the LOD score is indicated on Y‐axis. The horizontal red line represents LOD threshold of significance

We conducted a QTL analysis to detect genomic loci contributing to the observed variation in *H. armigera* resistance component traits in the RIL population. In the present study, a total of nine main‐effect QTLs (M‐QTLs, QTLs explaining more than 10% of the phenotypic variance) were identified for seven *H. armigera* resistance component traits (Figure 3; Table 3). A main‐effect QTL on CaLG01 was detected for unit larval weight measured at vegetative stage (*qUlwv01*), during 2008–2009 season, with a peak LOD score of 3.71. The *qUlwv01* QTL (peak marker Ca1_13535827) explains about 17.27% of the phenotypic variation observed in the mapping population and spans a distance of 13.81 cM. Additionally, M‐QTLs for unit larval weight at flowering stage (*qUlwf03*) and *H. armigera* larvae per 10 plants (*qLpp03*) were identified on CaLG03, with a peak LOD score of 4.56 and 4.89, respectively. Here, *qUlwf03* (peak marker Ca3_26424114) explains about 20.80% phenotypic variance and spans an interval of 82.85 cM, while *qLpp03* (peak marker Ca3_25961542) explains 22.14% of the phenotypic variance and spans an interval of 19.60 cM. The M‐QTLs for damage rating at vegetative stage (*qDrvl04*) evaluated during 2007–2008 season and damage rating at flowering stage (*qDrfl04*) were identified on CaLG04, with peak LOD scores of 6.31 and 3.00, respectively. Four M‐QTLs for flowering traits, namely *qDf03* (peak marker Ca3_26878028) on CaLG03 and *qDf07* (peak marker Ca7_9011339) on CaLG07 for days to first flowering, and *qD50f03* (peak marker Ca3_26878028) on CaLG03 and *qD50f07* (peak marker Ca7_9011339) on CaLG07 for days to 50% flowering, were identified. Considerable overlap in the confidence interval of the QTL peaks for both the traits was observed (Figure 3). Here, *qDf03* possesses a peak LOD score of 10.81 and explains up to 42.49% of the phenotypic variance, while *qDf07* possess a peak LOD score of 3.42 and explains about 16.06% of the phenotypic variance. In addition, *qD50f03* with a peak LOD score of 10.52 and *qD50f07* with a peak LOD score of 3.29 explain about 41.63% and 15.51% of the phenotypic variance, respectively. Interestingly, no minor QTL was observed for any of the above‐mentioned component traits.

**TABLE 3:** Summary of main‐effect QTLs identified for *H. armigera* resistance component traits using the genetic map for ICC 4958 × PI 489777 RIL population

Trait	QTL Id	Linkage group	Position (cM)	Peak marker	Left marker	Right marker	Left marker position (cM)	Right marker position (cM)	LOD value	PVE (%)
ULWV	*qUlwv01*	CaLG01	125.30	Ca1_13535827	Ca1_13607141	Ca1_12120720	124.70	138.51	3.71	17.27
ULWF	*qUlwf03*	CaLG03	12.45	Ca3_26424114	Ca3_24150445	Ca3_39192658	0.00	82.85	4.56	20.80
LPP	*qLpp03*	CaLG03	11.29	Ca3_25961542	Ca3_24150445	Ca3_27575961	0.00	19.60	4.89	22.14
DRV_L	*qDrvl04*	CaLG04	93.00	Ca4_12952156	Ca4_12952156	Ca4_37364166	82.96	240.72	6.31	27.59
DRF_L	*qDrfl04*	CaLG04	94.00	Ca4_17064735	Ca4_9847572	Ca4_37364166	65.95	240.72	3.00	14.05
DFF	*qDf03*	CaLG03	15.29	Ca3_26878028	Ca3_25230187	Ca3_27556951	6.18	19.29	10.81	42.49
DFF	*qDf07*	CaLG07	0.00	Ca7_9011339	Ca7_9011339	Ca7_4203236	0.00	28.05	3.42	16.06
D50F	*qD50f03*	CaLG03	15.29	Ca3_26878028	Ca3_25230187	Ca3_27556951	6.18	19.29	10.52	41.63
D50F	*qD50f07*	CaLG07	0.00	Ca7_9011339	Ca7_9011339	Ca7_4203236	0.00	28.05	3.29	15.51

*QTL*, quantitative trait locus; *cM*, centiMorgan; *LOD*, logarithm of odds; *PVE*, phenotypic variance explained.

**FIGURE 4:**
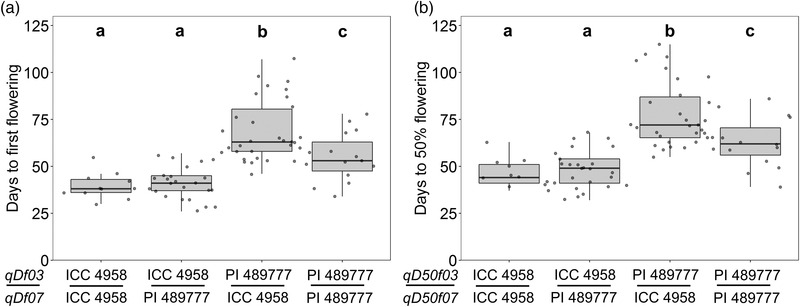
Boxplots indicating the joint allelic effects of two major loci on flowering time for plants grown under field conditions. (a) Phenotype by genotype plot for two main‐effect QTLs (*qDf03*, FT candidate and *qDf07*, FCA candidate) influencing days to first flowering in ICC 4958 × PI 489777 RIL population evaluated under field conditions, containing the simultaneous occurrence of both QTLs. (b) Phenotype by genotype plot for two major loci (*qD50f03*, FT candidate and *qD50f07*, FCA candidate) having an effect on days to 50% flowering in the mapping population, in which both loci are concomitantly present. The alphabets (a, b, c) above box plots designate statistical significance between the genotypes at *qDf03, qDf07, qD50f03*, and *D50f07*, computed using Tukey‐Kramer test (*p *< .05)

The data for flowering time was analyzed using a two‐QTL model, to identify the joint effects of *qDf03* and *qDf07* QTLs on days to first flowering, and of *qD50f03* and *qD50f07* QTLs on days to 50% flowering. Interestingly, ICC 4958 alleles at both *qDf03* and *qDf07* were linked with rapid flowering, while RILs with PI 489777 alleles at *qDf03* and *qDf07* usually had delayed flowering (Figure 4a). Despite this general trend, if a particular RIL possessed PI 489777 allele at *qDf03* and ICC 4958 allele at *qDf07*, this resulted in a substantial delay in flowering time, but with more variability. Furthermore, presence of ICC 4958 alleles at *qD50f03* and *qD50f07* were associated with a decrease in days to 50% flowering, whereas presence of PI 489777 alleles at both *qD50f03* and *qD50f07* were related to RILs having delayed flowering (Figure 4b). A line with a mix for PI 489777 allele at *qD50f03* and ICC 4958 allele at *qD50f07*, typically led to a remarkable increase in days to 50% flowering. While no other marked exceptions were observed in the described pattern, we predict the absence of additional major‐effect loci contributing to flowering time variation within this population.

### 3.5 Epistatic QTLs for combinations of two loci and three loci interactions

**FIGURE 5:**
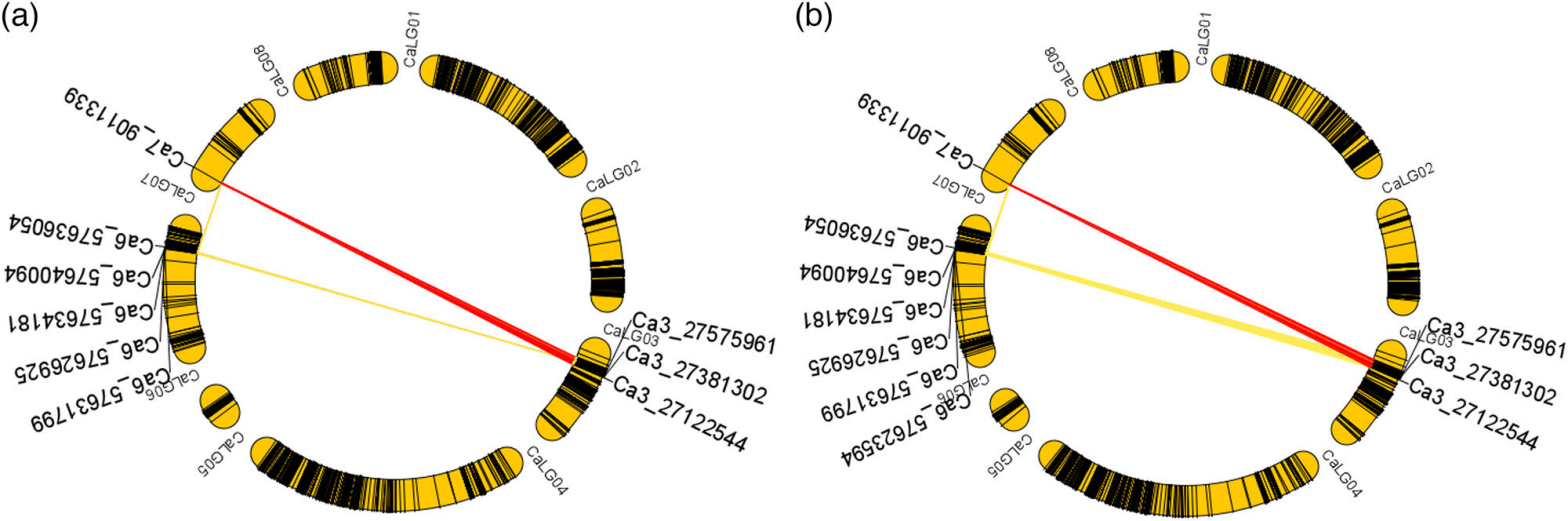
Graphical illustration of interacting triple loci and their locations on the chickpea genetic map detected by Genotype Matrix Mapping. (a) Depiction of interacting three loci together with their positions on the linkage map for days to first flowering. (b) Delineation of interacting triple loci combinations and their locational details on the genetic map for days to 50% flowering in the RIL population. Eight linkage groups of chickpea are represented in tandem arrangement as a circle. Triangles within the circle display interacting three loci combinations. The color of each line, varying from yellow to red, indicates the magnitude of QTL interaction

For simplifying the complexity of *H. armigera* resistance component traits, the genotype matrix mapping (GMM) program (Isobe, Nakaya, & Tabata, 2007) was utilized to identify QTL interactions in complex traits within the RIL population. Here, epistatic QTLs (E‐QTLs) were detected only for two flowering time traits, namely days to first flowering and days to 50% flowering. Interestingly, the results obtained using GMM analysis were consistent with those obtained using R/qtl. A total of 9 E‐QTLs were identified for two flowering traits, for combinations of two loci (Supplemental Table S4). The combinations of two interacting loci between CaLG03 and CaLG07 for days to first flowering and for days to 50% flowering explain up to 38.05% and 36.97% phenotypic variance, respectively. Additionally, 946 E‐QTLs were detected for combinations of three loci interactions. Figure 5 represents the combinations of interacting triple loci and their positions on the chickpea linkage map. The positions of interacting loci are connected by lines on linkage groups. Here, three loci interactions between CaLG07, CaLG03 and CaLG03 for days to first flowering explain 36.79–38.05% phenotypic variance; while those between CaLG07, CaLG03 and CaLG03, or CaLG07, CaLG06 and CaLG03 for days to 50% flowering explain 34.80–36.97% phenotypic variance.

### 3.6 Candidate genes underlying M‐QTLs for *H. armigera* resistance component traits

Based on the QTL analysis, we mined the potential candidate *H. armigera* resistance genes in chickpea, which co‐localized to the most important QTL peaks. For *qUlwv01*, a total of 749 candidate genes were predicted in the confidence interval spanning from 124.70 cM to 138.51 cM (Supplemental Table S5). The peak SNP marker for *qUlwv01* (Ca1_13535827) was linked to a MYB transcription factor *MYB48* (*Ca_14119*) gene. MYB transcription factors have played a crucial role in enhancing resistance against *H. armigera* in legumes. For instance, overexpression of R2R3‐MYB transcription factor from wild soybean in transgenic *Arabidopsis* plants has displayed high resistance against *H. armigera* larvae (Shen et al., 2018). Previous studies on plant lectins and flavonoids have shown adverse effects of these compounds on *H. armigera* larvae (Ohizumi et al., 2009; Sharma, Sharma, Seetharama, & Ortiz, 2000). Here, we predicted lectin receptor kinase (*Ca_14073*, *Ca_14087*, *Ca_14088*) and chalcone and stilbene synthase (*Ca_08294*) as the plausible candidate genes underlying *qUlwv01*, which might also influence larval weight in this mapping population. The mapping interval of *qUlwf03* spanning a region from 0.0 cM to 82.85 cM encompassed a total of 1,458 candidate genes (Supplemental Table S5). The peak marker for *qUlwf03*, Ca3_26424114, was localized in the vicinity of a FLOWERING LOCUS T (FT) protein (*Ca_08264*), which acts as a floral pathway integrator regulating floral development (Corbesier & Coupland, 2006). Proteinase inhibitors (PIs) are considered as direct defences that are induced by plants in response to insect attack (Kuwar, Pauchet, Vogel, & Heckel, 2015). Several laboratory‐scale studies have demonstrated the successful use of proteinase and α‐amylase inhibitors in controlling the growth and development of *H. armigera* larvae (Akbar, Jaba, Regode, Siva Kumar, & Sharma, 2018; Majidiani, Farshbaf Pour Abad, & Bandani, 2014; Tanpure et al., 2017). Here, two candidate genes, namely cysteine proteinase inhibitor 12‐like (*Ca_08112*) and legumin A‐like (*Ca_12231*), possessing cysteine‐type endopeptidase and α‐amylase inhibitor activity, respectively, co‐localized to *qUlwf03* peak.

The *qLpp03* mapping interval spanning from 0.0 cM to 19.60 cM, predicted the presence of 297 candidate genes (Supplemental Table S5). Salicylic acid carboxyl methyltransferase (SAMT) catalyzes the formation of methyl salicylate from salicylic acid (Ross, Nam, D'Auria, & Pichersky, 1999). It is predicted to be involved in a diverse array of defence responses (Xu, Song, & Zheng, 2006). The peak marker of *qLpp03* led to the prediction of salicylic acid carboxyl methyltransferase (*Ca_08292*), as a potential candidate gene regulating the number of pod borer larvae on chickpea plants. Although several genes lie within the estimated QTL interval, the closely linked resistance genes including peroxidase family (*Ca_08185*), ricin‐type beta‐trefoil lectin domain (*Ca_08256*) and chalcone and stilbene synthase family (*Ca_08294*) are likely candidates, as previous studies have shown that these genes play major roles in imparting defence against lepidopteran species (Dowd & Lagrimini, 1997). A total of 1,392 candidate genes were anticipated to be localized within the mapping interval of *qDrvl04* traversing from 82.96 cM to 240.72 cM (Supplemental Table S5). The peak marker of *qDrvl04* (Ca4_12952156) was linked to a protein of unknown function (*Ca_04516*). Additionally, 1,672 candidate genes were predicted in the confidence interval of *qDrfl04* spanning from 65.95 cM to 240.72 cM. Apart from a lectin receptor kinase (*Ca_14181*), no other candidate genes that were described to regulate *H. armigera* resistance in crop plants (Sharma et al., 2000), were found to co‐localize with *qDrvl04* and *qDrfl04* interval; thus providing an opportunity to scrutinize the effectors of the molecular control of damage caused by *H. armigera* in chickpea.

We also predicted candidate genes underlying *qDf03, qD50f03, qDf07* and *qD50f07*, which might influence flowering time in this mapping population. The *qDf03* and *qD50f03* mapping interval spanning from 6.18 cM to 19.29 cM comprised of 199 candidate genes (Supplemental Table S5). The plastocyanin‐like domain‐containing protein (*Ca_08221*), which was linked with *qDf03* and *qD50f03* peak marker, is predicted as a potential candidate gene. Additionally, the mapping interval of *qDf03* and *qD50f03* also contained previously identified candidate genes including FLOWERING LOCUS T (FT; *Ca_08264*) and nuclear‐cap binding protein (*Ca_23894*), which have been demonstrated to regulate flowering time (Corbesier et al., 2007; Kuhn, Breton, & Schroeder, 2007). The confidence interval for *qDf07* and *qD50f07* comprised a total of 523 candidate genes (Supplemental Table S5). Amidst several genes present in the confidence interval, the previously reported flowering response genes including flowering time control FCA (*Ca_03031*), flowering‐promoting factor (*Ca_06849*) and MYB‐like transcription factor family (*Ca_06627*) genes (Hoenicka et al., 2012; Macknight et al., 1997) are anticipated to control flowering in the present RIL population.

### 3.7 Candidate genomic region for molecular breeding for improving *H. armigera* resistance component traits

During the analysis of robust M‐QTLs, an interesting genomic region (∼ 20 cM) was detected on CaLG03. The intervals of M‐QTLs for three traits (LPP, DFF and D50F), which explain up to 42.49% phenotypic variance were found to co‐localize in this region (Table 3). Moreover, several E‐QTLs obtained for DFF and D50F were also associated with this genomic region (Supplemental Table S4). A total of 52 SNP markers were mapped in this genomic region. Taking into account the potential of this region in governing crucial pod borer resistance and flowering time traits, it can be considered as a promising candidate genomic region for molecular breeding of pod borer, *H. armigera* resistance in chickpea.

## 4 DISCUSSION

Molecular markers have been routinely used in breeding programs for trait dissection and mapping, evaluating genetic diversity, estimating evolutionary relationships, and marker‐assisted selection strategies (Roorkiwal et al., 2020). In general, QTL mapping (Roorkiwal et al., 2018; Sivashakthi et al., 2018; Soren et al., 2020) and association mapping (Varshney et al., 2012b, 2019) are used for identification of molecular marker(s) associated with the trait of interest. In the current study, we used a QTL mapping approach for an interspecific RIL population (ICC 4958 × PI 489777) for understanding the genetic architecture of *H. armigera* resistance component traits. Frequency distribution of all studied traits displayed characteristics of continuous variation, and revealed transgressive segregation in both directions, thereby suggesting the contribution of favorable alleles from both parents for these component traits. Understanding the interaction between genotypes and environments is crucial in crop improvement programs because a substantial genotype × environment interaction can greatly impair selection of superior genotypes in crop development efforts (Kang, Prabhakaran, & Mehra, 2004). Analysis of variance results suggest that both damage rating and larval weight evaluated during the vegetative phase of plant growth, in general, are affected by the environment in which the genotypes are evaluated.

The success of genomics‐assisted breeding (GAB) mainly depends on the level of marker precision and cost‐effectiveness of the technology used for genotyping. Array‐based platforms are capable of providing speedy high‐density genome‐wide scans, accurate allele calling together with good call rates, and enhanced cost‐efficiency when genotyping huge number of samples and SNP markers (Rasheed et al., 2018). High‐quality Axiom*CicerSNP* array containing 50,590 non‐redundant SNPs offers a unique opportunity to precisely undertake trait genetics and molecular breeding for chickpea improvement. In the present study, a total of 3,873 SNPs could be mapped out of 18,033 polymorphic markers. This may be due to either the stringent filtering criteria or use of an interspecific mapping population developed using wild progenitor as one of the parent, while Axiom*CicerSNP* array was developed using SNPs from the cultivated genome. A high marker density in the current genetic map will facilitate the selection of tightly linked markers for multiple molecular breeding applications in the future. Moreover, characterizing the RIL population permitted us to determine the genetic loci controlling *H. armigera* resistance component traits in chickpea. For a given component trait under specific conditions, only one or two M‐QTLs were identified, indicating that *H. armigera* resistance between ICC 4958 and PI 489777 is controlled by a limited number of genes with large effects.

The magnitude of damage caused during pod filling stage can be minimized by selecting genotypes that flower before or after the peak abundance of *H. armigera*, thereby, suffering less damage than those flowering during the episodes of utmost insect abundance (Kambrekar, Balikai, Giraddi, Aruna, & Neelakanth, 2016). Several studies have mapped major‐ and minor‐effect QTLs for flowering time and vernalization response on CaLG03 in chickpea (Daba, Deokar, Banniza, Warkentin, & Tar'an, 2016; Gaur, Samineni, Tripathi, Varshney, & Gowda, 2014; Jamalabadi, Saidi, Karami, Kharkesh, & Talebi, 2013; Mallikarjuna et al., 2017; Samineni, Kamatam, Thudi, Varshney, & Gaur, 2016). Our results on flowering time QTLs (*qDf03* and *qD50f03*) identified on CaLG03 also support findings from these studies. Interestingly, *qDf07* and *qD50f07* identified on CaLG07 in the current study have not been reported previously, and seems to be novel genetic loci controlling flowering time in chickpea.

Identification of potential genes underlying the QTL interval is important for practical crop breeding. We predicted the plausible candidate genes underlying the mapped QTLs for *H. armigera* resistance component traits in chickpea. Plant lectins constitute a heterogeneous group of sugar binding proteins, which have been previously reported to affect the survival and growth of insect pests (Gilardoni, Hettenhausen, Baldwin, & Bonaventure, 2011; Sharma et al., 2000). In the present study, candidate genes belonging to lectin receptor kinase and chalcone and stilbene synthase family, underlying *qUlwv01* are predicted to alter the weight of pod borer larvae feeding on vegetative stage chickpea leaves. Inhibition of cysteine proteinase and α‐amylase have been shown to reduce the performance of insect and virus in barley, rice and *Arabidopsis* (Carrillo et al., 2011; Gadge et al., 2015; Leplé et al., 1995). Our findings complement previous reports and also suggest that cysteine proteinase inhibitor 12‐like and legumin A‐like genes may inhibit cysteine proteinase and α‐amylase activity, respectively, in the midgut of *H. armigera*, thereby controlling its weight.

A previously identified flowering‐time (FT) gene was found to co‐localize with the *qDf03* and *qD50f03* mapping interval. Synthesis of FT, a small and mobile protein, in leaves followed by its transmission to shoot apex induces flowering in *Arabidopsis thaliana* and many other plant species (Corbesier et al., 2007). Interestingly, higher expression of FT gene homologs was associated with the early‐flowering phenotype of ICCV 96029 in chickpea (Ridge et al., 2017). Plastocyanin (PC) is a copper‐containing soluble protein that is localized in the thylakoid lumen, which mediates the transfer of electrons to photosystem I (Weigel et al., 2003). Analysis of the early flowering mutant *sucrose uncoupled2* (*sun2*), identified by reduced repression of a transgenic PC promoter by sucrose (Dijkwel, Huijser, Weisbeek, Chua, & Smeekens, 1997), predicted a plausible relationship between PC and flowering time control in plants. All together, we predicted 19 potential candidate genes underlying nine M‐QTLs, which hold the promise to impact *H. armigera* resistance component traits in chickpea.

## 5 CONCLUSION

We evaluated the RIL population to dissect the genetic architecture of *H. armigera* resistance component traits in chickpea. A dense genetic map developed in this study will be immensely useful for genetic dissection of other plant traits, such as seed yield, plant height, shoot biomass and harvest index, which differ between ICC 4958 and PI 489777 genotypes. We detected nine M‐QTLs that control pod borer resistance component traits, such as damage rating, larval weight, number of larvae per 10 plants, larval survival percentage, pod damage percentage and flowering time, amongst others. These QTLs possess a LOD score ranging from 3.00–10.81 and explain 14.05–42.49% phenotypic variance. Several of these QTLs were found to co‐localize with previously predicted *H. armigera* resistance and flowering time genes, revealing evolutionary upkeep of molecular mechanisms between domesticated and wild species. Notably, few QTLs did not overlap with previously described genes, thus offering a unique opportunity to expand our knowledge about the molecular control of *H. armigera* resistance component traits in chickpea. However, further fine‐mapping and functional characterization of these genes is required to explicitly pinpoint the candidate genes underlying the identified QTLs.

## CONFLICT OF INTEREST

The authors declare that they have no conflict of interest.

## AUTHOR CONTRIBUTIONS

MR and RKV conceived the idea and provided critical inputs to the concept. HCS together with JJ, SPS and RM coordinated generation and analysis of phenotyping data. MR, AC and RKV contributed in genotyping data generation. RB analysed the phenotyping and genotyping data for constructing genetic map and QTL mapping analysis. RB drafted sections of the manuscript and prepared tables and figures. RB, JJ, SRS, MR, HCS and RKV made a critical revision of the content of the manuscript. All authors contributed to the final reading and approved the submitted version. Rutwik Barmukh: Data curation; Formal analysis; Writing‐original draft. Manish Roorkiwal: Conceptualization; Formal analysis; Project administration; Supervision; Writing‐review & editing. Jagdish Jaba: Data curation; Methodology; Resources. Annapurna Chitikineni: Methodology; Resources. Suraj Prasad Mishra: Data curation; Methodology. Someswar Rao Sagurthi: Methodology; Supervision. Rajendra Munghate: Data curation; Methodology. HC Sharma: Data curation; Methodology; Project administration; Resources; Writing‐review & editing. Rajeev K. Varshney: Conceptualization; Funding acquisition; Project administration; Resources; Supervision; Writing‐review & editing

## Supporting information

Supporting Material

Supporting Material

Supporting Material

Supporting Material

Supporting Material

Supporting Material
