# Supplementary material for: Development of a dense genetic map and QTL analysis for pod borer Helicoverpa armigera (Hübner) resistance component traits in chickpea (Cicer arietinum L.)
Source: Plant Genome. 2020 Dec 1;14(1):e20071. doi: 10.1002/tpg2.20071 (PMC12807004; doi:10.1002/tpg2.20071)
Supplement: Supplementary file 1 [file TPG2-14-e20071-s001.docx]

**Development of a dense genetic map and QTL analysis for pod borer *Helicoverpa armigera* (Hübner) resistance component traits in chickpea (*Cicer arietinum* L.)**

Rutwik Barmukh^1,2^, Manish Roorkiwal^1,*^, Jagdish Jaba^3^, Annapurna Chitikineni^1^, Suraj Prasad Mishra^3^, Someswar Rao Sagurthi^2^, Rajendra Munghate^3^, HC Sharma^3,*^, Rajeev K. Varshney^1,*^

^1^Center of Excellence in Genomics & Systems Biology, International Crops Research Institute for the Semi-Arid Tropics (ICRISAT), Hyderabad, India

^2^Department of Genetics, Osmania University, Hyderabad, India

^3^Theme- Integrated Crop Management, International Crops Research Institute for the Semi-Arid Tropics (ICRISAT), Hyderabad, India

*Correspondence:

**Manish Roorkiwal** and **Rajeev K. Varshney**: Center of Excellence in Genomics & Systems Biology, International Crops Research Institute for the Semi-Arid Tropics (ICRISAT), Hyderabad, India.

**HC Sharma:** Theme- Integrated Crop Management, International Crops Research Institute for the Semi-Arid Tropics (ICRISAT), Hyderabad, India

E-mail addresses: [m.roorkiwal@cgiar.org](mailto:m.roorkiwal@cgiar.org) (Manish Roorkiwal), [hcsh19@gmail.com](mailto:hcsh19@gmail.com) (HC Sharma) and [r.k.varshney@cgiar.org](mailto:r.k.varshney@cgiar.org) (Rajeev K. Varshney).

**Supplemental Figures**

**Supplemental Figure S1. Histograms of distribution for *H. armigera* resistance component traits evaluated using detached leaf assay in the RIL population.** Traits measured during different seasons include: (a) Damage rating at vegetative stage during 2007-08 season (DRV_L | 2007-08), (b) Damage rating at vegetative stage during 2008-09 season (DRV_L | 2008-09), (c) Damage rating at flowering stage during 2007-08 season (DRF_L | 2007-08), (d) unit larval weight at vegetative stage during 2007-08 season (ULWV | 2007-08), (e) unit larval weight at vegetative stage during 2008-09 season (ULWV | 2008-09), (f) unit larval weight at flowering stage during 2007-08 season (ULWF | 2007-08), and (g) larval survival percentage during 2008-09 season (LSP | 2008-09). Blue arrow indicates the average phenotypic value for ICC 4958 plants and red arrow indicates the average phenotypic value for PI 489777 plants.

**Supplemental Figure S2. Histograms of distribution for *H. armigera* resistance component traits evaluated under field conditions.** Traits measured during different seasons include: (a) damage rating at vegetative stage during 2007-08 season (DRV_F | 2007-08), (b) damage rating at vegetative stage during 2008-09 season (DRV_F | 2008-09), (c) pod damage percentage during 2008-09 season (PDP | 2008-09), (d) larvae per 10 plants during 2007-08 season (LPP | 2007-08), (e) number of larvae per plant at vegetative stage during 2008-09 season (NLV | 2008-09), (f) number of larvae per plant at flowering stage during 2008-09 season (NLF | 2008-09), (g) days to first flowering during 2008-09 season (DFF | 2008-09), and (h) days to 50% flowering during 2008-09 season (D50F | 2008-09). Blue arrow indicates the average phenotypic value for ICC 4958 plants and red arrow indicates the average phenotypic value for PI 489777 plants.

**Supplemental Figure S3. Filtration of the data for prioritizing SNP markers and RILs included in the analysis.** The results obtained for different steps that were performed during data filtration, have been displayed (a-f).

**Supplemental Figure S4. Comparison of the physical map and genetic map for ICC 4958 × PI 489777 mapping population.** (a) Physical location (Mb) of filtered SNP markers along the chickpea linkage groups. (b) Genetic map of selected SNP markers. *Mb*, megabase; *cM*, centiMorgan.
